# Heterogeneity amongst GLP-1 RA cardiovascular outcome trials results: can definition of established cardiovascular disease be the missing link?

**DOI:** 10.1186/s13098-021-00698-5

**Published:** 2021-07-27

**Authors:** Miguel Melo, Cristina Gavina, José Silva-Nunes, Luís Andrade, Davide Carvalho

**Affiliations:** 1 Department of Endocrinology, Diabetes and Metabolism, Centro Hospitalar e Universitário de Coimbra, Praceta, R. Prof. Mota Pinto, 3004-561 Coimbra, Portugal; 2 Faculty of Medicine, Universidade de Coimbra, Coimbra, Portugal; 3 Cardiology Department, Hospital Pedro Hispano-ULS Matosinhos, Matosinhos, Portugal; 4 Cardiovascular Research and Development Unit, Faculty of Medicine, Universidade do Porto, Porto, Portugal; 5 Department of Endocrinology, Diabetes, and Metabolism, Centro Hospitalar Universitário de Lisboa Central, Lisboa, Portugal; 6 NOVA Medical School / Faculdade de Ciências Médicas, Universidade Nova de Lisboa, Lisboa, Portugal; 7 Health and Technology Research Center (H&TRC), Escola Superior de Tecnologia da Saúde de Lisboa, Lisboa, Portugal; 8 Centro Hospitalar de Vila Nova de Gaia-Espinho, Vila Nova de Gaia, Portugal; 9 Department of Endocrinology, Diabetes and Metabolism, Centro Hospitalar E Universitário S. João, Porto, Portugal; 10 Faculty of Medicine, Universidade do Porto, Porto, Portugal; 11 i3SInstituto de Investigação e Inovação Em Saúde, Universidade do Porto, Porto, Portugal

**Keywords:** Antidiabetic drug, GLP-1 RA, Cardiovascular disease, Cardiovascular outcome trials, Type 2 diabetes

## Abstract

Atherosclerotic cardiovascular diseases are the leading cause of adverse outcomes in patients with type 2 diabetes, and all new anti-diabetic agents are mandated to undergo cardiovascular outcome trials (CVOTs). Glucagon-like peptide-1 receptor agonists (GLP-1 RA) are incretin mimetics that reduce blood glucose levels with a low associated risk of hypoglycaemia. CVOTs with different GLP-1 RAs yielded different results in terms of major cardiovascular composite outcome (MACE), with some trials showing superiority in the treatment arm, whereas other simply displayed non-inferiority. More importantly, the significance of each component of MACE varied between drugs. This begs the question of whether these differences are due to dissimilarities between drugs or other factors, namely trial design, are at the root of these differences. We analyse the trial designs for all CVOTs with GLP-1 RAs and highlight important differences between them, namely in terms of definition of established cardiovascular disease, and discuss how these differences might explain the disparate results of the trials and preclude direct comparisons between them. We conclude that a fair comparison between GLP-1 RA CVOTs would involve post-hoc analysis re-grouping the patients into different cardiovascular risk categories based upon their baseline clinical parameters, in order to even out the criteria used to classify patients.

## Background

Diabetes is one of the most prevalent chronic diseases worldwide. According to the 2019 International Diabetes Federation report, global diabetes prevalence, between 20 and 79 years of age, is estimated to be 9.3% (463 million people) and is projected to increase by 25% in 2030 and 51% in 2045 [1].

Diabetes is a devastating disease, associated with an ominous prognosis. Diabetes relative mortality rates were reported to be approximately twice to 4 times as high compared with individuals without diabetes, with an estimated 4.2 million deaths directly caused by the disease or by its complications in 2019, almost half of them before the age of 60 years [1]. Diabetes is also a major cause of morbidity. Amongst diabetes-related complications, atherosclerotic cardiovascular diseases (ASCVD) remain the leading cause of mortality and adverse outcomes in patients with type 2 diabetes mellitus (T2D) [2]. Events of ASCVD in diabetes mellitus include coronary heart disease, ischemic stroke, peripheral artery disease (PAD), and heart failure (HF) [2]. These manifestations generally occur earlier and are more severe and diffuse than in non-diabetic patients. Moreover, according to the Emerging Risk Factors Collaboration data, their combination has a multiplicative effect on mortality risk [3].

Even though in recent years the incidence of diabetes-related cardiovascular (CV) complications in T2D patients have been decreasing, these remain higher than in non-diabetics [4–6]. The control of five risk factors for cardiovascular disease in T2D patients–elevated glycated haemoglobin (HbA1c) level, elevated low-density lipoprotein cholesterol level, albuminuria, smoking, and elevated blood pressure – to within normal range brings almost equals the risks of death, stroke, and myocardial infarction of the general population. In a cohort study including 271,174 patients with T2D and matched with 1,355,870 controls, the risk of hospitalization for HF was consistently higher among patients with diabetes than among controls. A HbA1c level outside the target range was the strongest predictor of stroke and acute myocardial infarction [5]. We can assume that evidence-based secondary prevention therapies, although effective, are not able to close this gap between diabetics and non-diabetics, despite optimal glycemic control [7–9]. Likewise, coronary revascularization has worse outcomes in the presence of diabetes, with higher rates of stent thrombosis in percutaneous revascularization and worse survival after coronary artery bypass grafting (CABG), particularly in insulin treated patients [10, 11]. In multivessel disease, CABG has better results than percutaneous coronary intervention (PCI) with drug-eluting stents in preventing the occurrence of non-fatal myocardial infarction (MI), non-fatal stroke or all-cause death [12] and lower rates of all-cause death at a median follow-up of 7.5 years [13].

The risk of CV events is associated with hyperglycemia, and is increased even with glycemic levels below the threshold for diabetes, although this association is stronger for microvascular complications [14]. Paradoxically, therapeutic strategies used for intensive glycemic control have failed to prove beneficial in preventing major adverse CV events (MACE) in comparison to less stringent strategies, as shown by the results of the ACCORD (Action to Control Cardiovascular Risk in Diabetes) [15] and the ADVANCE (Action in Diabetes and Vascular Disease: Preterax and Diamicron Modified Release Controlled Evaluation) [16] and VADT (Veterans Affairs Diabetes Trial) [17] trials in 2008. In the ACCORD trial a significant increase of mortality that led to interruption of the study was observed, but that seem to be due to significant glycemic variability as those who experienced mortality didn't have a significant reduction in HbA1c despite forced titration of their therapy. Insulin and sulfonylureas were also used in that study. These studies used the glucose-lowering drugs available at the time, including drugs from pharmacological classes classically associated with a significant risk for hypoglycemia. In the light of new CV outcomes trials (CVOTs) results, we should revise our appraisal of the ACCORD and ADVANCE trials. Perhaps the focus should not be only on achieving the lowest glycemic levels, but rather on the mechanisms by which glycemic lowering is attained and collateral metabolic benefits obtained. Moreover, the non-glycemic effects of these new drug classes, impacting blood pressure and renal damage, may add to their beneficial effect on cardiovascular events.

Although the majority of diabetes-associated deaths in the ACCORD and ADVANCE trials were CV and the association of glycemic levels with MACE was only modest, it is interesting to see how the focus of previous trials with diabetic drugs was glucose lowering and not CV outcomes. Despite the positive outcomes of metformin use in the United Kingdom Prospective Diabetes Study (UKPDS) [18], it was a meta-analysis of trials with rosiglitazone, showing an increase in the rate of MI and a numerical increase in all-cause death [19], that alarmed regulatory agencies and, since 2008, prompted safety trials evaluating CV outcomes for new glucose-lowering agents.

Glucagon-like peptide-1 receptor agonists (GLP-1 RA) are glucose-lowering drugs used in the treatment of T2D, which carry low risk for hypoglycemia and induce weight loss [20]. CVOTs with GLP-1 RA included a variable number of patients with established CV diseases (eCVD) or at high risk for CV events, with different inclusion criteria (Tables 1 and 2). These differences make comparisons between trials impossible and may have influenced trial results. For this reason, we found it pertinent to review the different CVOTs performed with GLP-1 RA and perform a comparative analysis between them, highlighting the different study designs for each one.

**Table 1:** Trial design

	ELIXA [21]	LEADER [23]	SUSTAIN-6 [25]	EXSCEL [26]	Harmony Outcomes [29]	PIONEER 6 [31]	REWIND [33]
NCT number	NCT01147250	NCT01179048	NCT01720446	NCT01144338	NCT02465515	NCT02692716	NCT01394952
Drug	Lixisenatide	Liraglutide	Semaglutide	Exenatide	Albiglutide	Semaglutide	Dulaglutide
Posology	Daily Subcutaneous	Daily Subcutaneous	Once-weekly Subcutaneous	Once-weekly Subcutaneous	Once-weekly Subcutaneous	Daily Oral	Once-weekly Subcutaneous
Trial phase	III (pre-approval)	IIIb (post-approval)	III (pre-approval)	III/IV (post-approval)	IV (post-approval)	III (pre-approval)	III/IV (post-approval)
Primary analysis	Non-inferiority (upper range of the two-sided 96% CI < 1.3)	Non-inferiority (upper range of the two-sided 95% CI < 1.3)	Non-inferiority (upper limit of the 95% CI < 1.8)	Superiority (upper limit of the 95% CI is < 1.00)	Non-inferiority (upper range of the two-sided 95% CI < 1.3)	Non-inferiority (upper limit of the 95% CI < 1.8)	Superiority
Primary outcome	4-point MACE: CV death, MI, stroke, or hospitalization for UA	3-point MACE: CV death, MI, or stroke	3-point MACE: CV death, MI, or stroke	3-point MACE: CV death, MI, or stroke	3-point MACE: CV death, MI, or stroke	3-point MACE: CV death, MI, or stroke	3-point MACE: CV death, MI, or stroke

*CI* confidence interval, *CV* cardiovascular, *MACE* major adverse cardiovascular outcome, *MI* myocardial infarction, *UA* unstable angina

**Table 2:**
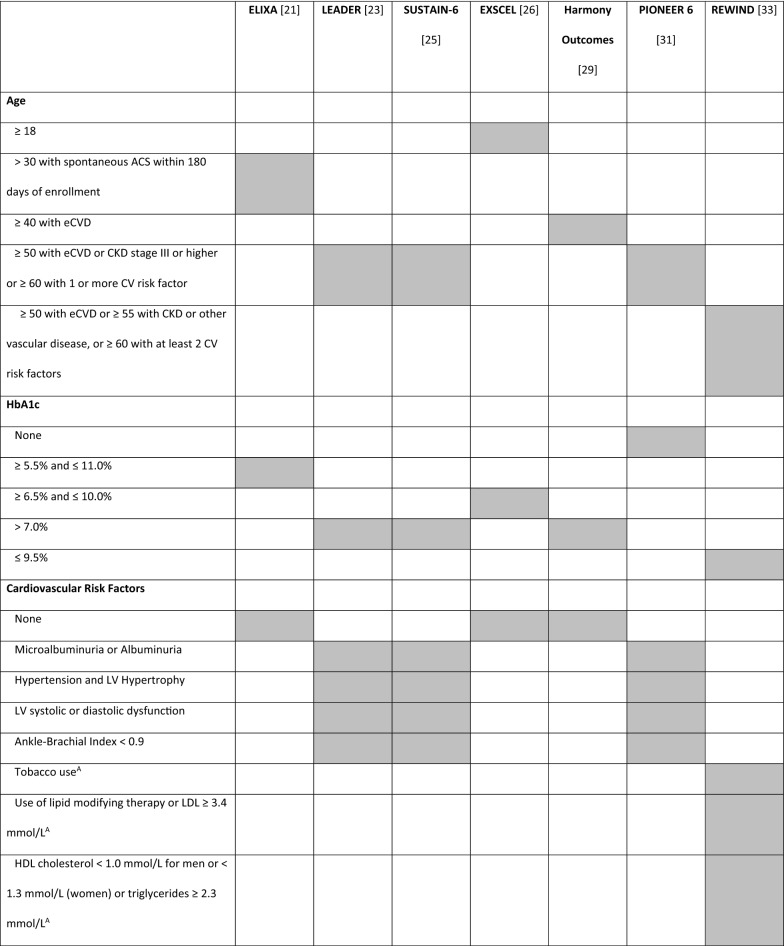

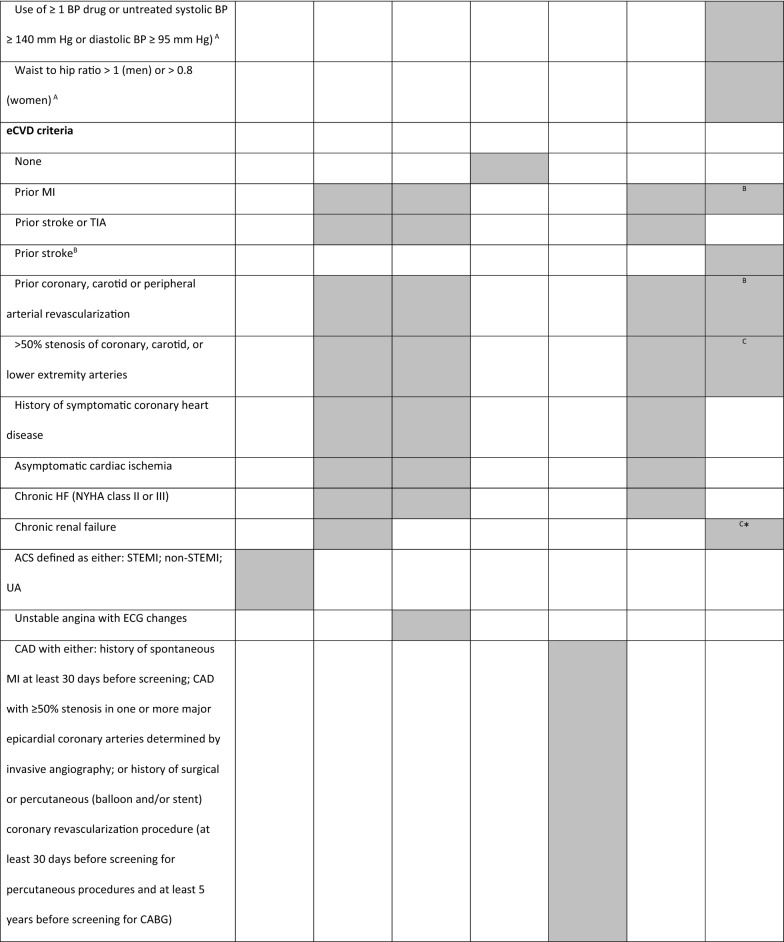

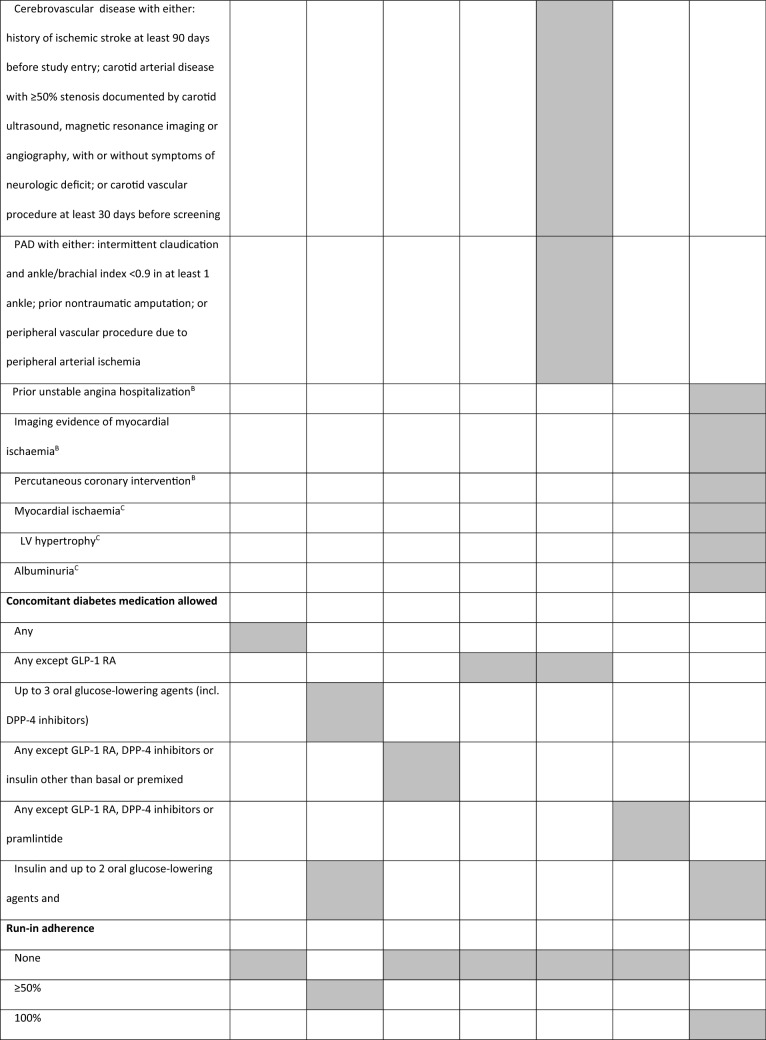
Trial key inclusion criteria

*ACS* acute coronary syndrome, *BP* blood pressure, *CAD* coronary artery disease, *CKD* chronic kidney disease, *CV* cardiovascular, *ECG* electrocardiogram, *eCVD * established cardiovascular disease, *HbA1c* haemoglobin A1c (glycated haemoglobin), *HDL* high-density lipoprotein, *HF* heart failure, *LDL* low-density lipoprotein, *LV* left ventricle, *MI* myocardial infarction, *NYHA* New York Heart Association classification, *PAD* peripheral artery disease, *STEMI* segment elevation myocardial infarction, *TIA* transient ischemic attack, UA unstable angina, *Grey shade* inclusion criteria adopted in the trialA – age 50–54 only; B—≥ 50 years; C – 55–59 years; *estimated glomerular filtration rate < 60 mL per minute per 1.73 m^2^

## Main characteristics of the GLP-1 RA CVOTs

### ELIXA

The Evaluation of Lixisenatide in Acute Coronary Syndrome (ELIXA) trial [21, 22] was a multicentre, randomized, double-blind, placebo-controlled trial to assess the effects of daily subcutaneous lixisenatide on CV outcomes in patients with T2D who had had a recent acute coronary event.

Patients with T2D were identified for a spontaneous acute coronary syndrome (ACS) within 180 days following hospitalization. ACS was defined as either segment elevation myocardial infarction (STEMI), non-STEMI, or unstable angina (UA). Inclusion criteria also required elevation of at least one cardiac biomarker (troponin or creatine kinase-MB isoenzyme).

The primary endpoint in the time-to-event analysis was a composite for a first episode of any of the following: death from CV causes, nonfatal MI, nonfatal stroke, or hospitalization for UA. This was the only of the analyzed trials to include UA as part of the primary endpoint, thus having a 4-point MACE primary outcome.

The ELIXA trial included 6068 patients, all of which had eCVD at baseline if one considers the ACS criteria, and who were followed for a relatively short time (2.1 years average follow-up period).

### LEADER

The Liraglutide Effect and Action in Diabetes: Evaluation of Cardiovascular Outcome Results (LEADER) trial [23, 24] was a multicenter, double-blind, placebo-controlled trial designed to assess the long-term effects of subcutaneous once-daily liraglutide on CV outcomes and other clinically important events in patients with T2D who were at high risk for CV disease (CVD). It was a post-approval trial designed to test primarily for non-inferiority, and thus the non-inferiority margin of the upper limit of the 95% Confidence Interval (CI) for MACE (primary outcome) was set at 1.3.

Two groups of patients were considered for enrolment: a) patients aged ≥ 50 years with eCVD [defined as: prior MI; prior stroke or transient ischemic attack; prior coronary, carotid or peripheral arterial revascularization; > 50% stenosis of coronary, carotid, or lower extremity arteries; history of symptomatic coronary heart disease documented by positive exercise stress test or any cardiac imaging or unstable angina with electrocardiographic (ECG) changes; asymptomatic cardiac ischemia documented by positive nuclear imaging test, exercise test or dobutamine stress echo; chronic HF [New York Heart Association (NYHA) class II or III)] or chronic renal failure [defined as an estimated glomerular filtration rate (eGFR) of less than 60 mL per minute per 1.73 m^2^], and b) patients aged ≥ 60 years with at least one high CVD risk factor (presence of microalbuminuria or proteinuria; hypertension and left ventricular hypertrophy by ECG or imaging; left ventricular systolic or diastolic dysfunction by imaging; ankle-brachial index < 0.9).

A total of 9340 patients were enrolled, 81.3% of which had eCVD at baseline, and were followed for an average of 3.8 years.

### SUSTAIN-6

The Trial to Evaluate Cardiovascular and Other Long-term Outcomes with Semaglutide in Subjects with Type 2 Diabetes (SUSTAIN-6) [25] was a pre-approval study designed to evaluate the non-inferiority of once-weekly subcutaneous semaglutide, as compared with placebo, in terms of CV safety. Being a pre-approval trial, the non-inferiority margin of the upper limit of the 95% CI for MACE was set at 1.8; for this reason, the sample size (N = 3297) is smaller than the samples of major post-approval CVOTs with antidiabetic drugs (as can be appreciated from Tables 1 and 3).

**Table 3:** Baseline population characteristics and primary outcome results

	ELIXA [22]	LEADER [24]	SUSTAIN-6 [25]	EXSCEL [27, 28]	Harmony Outcomes [30]	PIONEER 6 [32]	REWIND [34]
N	6068	9340	3297	14,752	9463	3183	9901
Female	30.7%	35.7%	39.3%	38.0%	30.6%	31.6%	46.3%
Age (mean)	60.3 years	64.2 years	64.6 years	62.7 years	62.7 years	66.1 years	66.2 years
HbA1c (mean)	7.7%	8.7%	8.7%	8.0%	8.7%	8.2%	7.2%
Diabetes duration	9.3 years	12.8 years	13.9 years	12.0 years	13.8 years	14.9 years	9.5 years
eCVD (according to each study’s definition)	100% (ACS)	81.3%	83.0%	73.1%	100%	84.6%	31.5%
HF (NYHA I-III)		17.8%	23.6%	16.2%	20.2%		8.6%
CKD (eGFR < 60 mL/min/1.73 m^2^)	23.2%	24.7%	28.5%	18.6%	22.6%	26.9%	22.2%
Follow-up (median)	2.1 years	3.8 years	2.1 years	3.2 years	1.5 years	1.3 years	5.4 years

*CKD* chronic kidney disease, *eCVD* established cardiovascular disease, *eGFR* estimated glomerular filtration rate, *HbA1c* haemoglobin A1c (glycated haemoglobin), *HF* heart failure, *NYHA* New York Heart Association classification

Key inclusion criteria were age ≥ 50 years with presence of eCVD or presence of chronic kidney disease stage 3 or higher (eGFR less than 60 mL per minute per 1.73 m^2^), or age ≥ 60 years and the presence of an additional CV risk factor. Established CVD included previous CV, cerebrovascular disease or PAD, and was defined according to the following criteria: prior MI, prior stroke or prior transient ischemic attack, prior coronary, carotid or peripheral arterial revascularization, more than 50% stenosis on angiography or imaging of coronary, carotid or lower extremities arteries, history of symptomatic coronary heart disease (documented by positive exercise stress test or any cardiac imaging) or unstable angina with ECG changes, asymptomatic cardiac ischemia (documented by positive nuclear imaging test or exercise test or stress echo or any cardiac imaging) or chronic HF NYHA class II-III. In the group of patients without eCVD, selection criteria based on the presence of risk factors included: persistent microalbuminuria (30‒299 mg/g) or proteinuria, hypertension and left ventricular hypertrophy (by ECG or imaging), left ventricular systolic or diastolic dysfunction by imaging, ankle/brachial index less than 0.9.

Overall, 83.0% of patients had eCVD, but all were at high CV risk, and patients were followed for a relatively short time (2.1 years) (Table 3), raising questions on whether these results would be generalizable for a more diverse population or for longer period of treatment [25].

### EXSCEL

EXSCEL (EXenatide Study of Cardiovascular Event Lowering trial) [26–28] was a multinational, placebo-controlled, double-blind, parallel-group pragmatic trial which randomized patients with T2D to receive once-weekly subcutaneous exenatide or matching placebo (unloaded microspheres), in addition to their usual care. It assessed the impact of once-weekly exenatide therapy in 14,752 patients, with or without known CVD.

The EXSCEL trial population was adults with T2D, with or without additional CV risk factors or prior CV events, and randomization targeted approximately 70% with a history of a cardiovascular event and 30% without known CVD, followed for 3.2 years on average.

A prior CV event was defined as at least one of the following: a) history of a major clinical manifestation of coronary artery disease, i.e., MI, surgical or percutaneous (balloon and/or stent) coronary revascularization procedure, or coronary angiography showing at least one stenosis ≥ 50% in a major epicardial artery or branch vessel, b) ischemic cerebrovascular disease, including: history of ischemic stroke (strokes not known to be hemorrhagic will be allowed as part of this criterion; transient ischemic attacks are not included), history of carotid arterial disease with ≥ 50% stenosis documented by carotid ultrasound, magnetic resonance imaging, or angiography, with or without symptoms of neurologic deficit, or c) atherosclerotic peripheral arterial disease, as documented by objective evidence such as amputation due to vascular disease, current symptoms of intermittent claudication confirmed by an ankle-brachial pressure index or toe-brachial pressure index less than 0.9, or history of surgical or percutaneous revascularization procedure.

Being a pragmatic trial, EXSCEL aimed to assess the effectiveness of exenatide in a setting similar to the real world, with broad inclusion criteria and streamlined procedures. It allowed a broad range of concomitant antidiabetic drugs, including DPP-4 inhibitors, which act on the same incretin system as GLP-1 RA. There was also no run-in period to screen for adherence, again making it more akin to a real world setting.

### Harmony outcomes

Harmony Outcomes (brief title: Effect of Albiglutide, When Added to Standard Blood Glucose Lowering Therapies, on Major Cardiovascular Events in Subjects With Type 2 Diabetes Mellitus) [29, 30] was a randomized, double-blind, placebo controlled trial of the effect of albiglutide on MACE in patients with T2D and established CVD. The trial enrolled 9463 participants aged ≥ 40 years with T2D, prior ASCVD, and suboptimal glycemic control. Participants were assigned in a 1:1 ratio to albiglutide 30 mg (potentially increasing to 50 mg) or matching placebo, administered once weekly by subcutaneous injection. The trial continued until 766 confirmed primary outcome events (cardiovascular death, MI, or stroke) occurred, over an average follow-up of 1.5 years.

The inclusion criteria were age ≥ 40 years with a diagnosis of T2D, eCVD, and HbA1c > 7.0% (53 mmol/mol). Established CVD was defined as at least one of the following: a) coronary artery disease with either: documented history of spontaneous MI, at least 30 days before screening; documented coronary artery disease with ≥ 50% stenosis in one or more major epicardial coronary arteries, determined by invasive angiography; or history of surgical or percutaneous (balloon and/or stent) coronary revascularization procedure (at least 30 days before screening for percutaneous procedures and at least 5 years before screening for CABG), b) cerebrovascular disease with either: documented history of ischemic stroke at least 90 days before study entry; carotid arterial disease with ≥ 50% stenosis documented by carotid ultrasound, magnetic resonance imaging or angiography, with or without symptoms of neurologic deficit; or carotid vascular procedure (e.g., stenting or surgical revascularization), at least 30 days before screening, or c) PAD with either: intermittent claudication and ankle/brachial index < 0.9 in at least 1 ankle; or prior non-traumatic amputation, or peripheral vascular procedure (e.g., stenting or surgical revascularization), due to peripheral arterial ischemia.

Besides the ELIXA, in which all patients had a previous ACS (see above), the Harmony Outcomes was the only CVOT in which the entire population had eCVD.

### PIONEER 6

The CV safety of the oral formulation of semaglutide was evaluated in the PIONEER 6 randomized controlled trial (official title: A Trial Investigating the Cardiovascular Safety of Oral Semaglutide in Subjects With Type 2 Diabetes) [31, 32]. This was also a pre-approval non-inferiority trial that included 3183 patients and demonstrated the CV safety of the drug. Inclusion criteria and primary outcome were exactly the same used in the SUSTAIN-6 trial [25] (Table 1 and 2), and 84.6% of the patients were selected due to the presence of CV or chronic renal disease (stage 3 or above—eGFR less than 60 mL per minute per 1.73 m^2^). Patients were followed for an even shorter time (1.3 years) than in the SUSTAIN-6 trial.

### REWIND

The Researching cardiovascular Events with a Weekly INcretin in Diabetes (REWIND) trial [33, 34] was a post-approval study designed to evaluate if the addition of once-weekly subcutaneous dulaglutide to standard of care was able to safely reduce the incidence of MACE in people with T2D aged ≥ 50 years. As in SUSTAIN-6, patient selection was based in a mixture of characteristics including age, presence of CVD and presence of risk factors. However, the selection of patients was more complex and included three subgroups: patients aged ≥ 50 years with eCVD [i.e., a previous MI, ischemic stroke, revascularization (coronary, carotid or peripheral), hospital admission for unstable angina, or imaging evidence of myocardial ischemia]; patients aged ≥ 55 years with subclinical CVD (defined by the presence of myocardial ischemia, coronary, carotid, or lower extremity artery stenosis exceeding 50% or left ventricular hypertrophy), eGFR less than 60 mL/min per 1.73 m^2^, or albuminuria; patients aged ≥ 60 years with two additional risk factors among tobacco use, dyslipidemia, hypertension, or abdominal obesity. However, when considering the groups with and without CV disease for reporting the results, patients were categorized as having CV disease if they had corresponded to the first group, with previous history of MI, ischemic stroke, unstable angina with ECG changes, myocardial ischemia on imaging or stress test, or coronary, carotid, or peripheral revascularization. Using the latter definition, 31.5% of the included population was considered to have CVD, making it the GLP-1 RA CVOT with the lowest percentage of eCVD population (Table 3). Nonetheless, as noted above (and systematized in Table 2), the criteria for eCVD in this trial differ somehow from the other CVOTs. The REWIND trial also included the highest percentage of females (46.3%), the population with the average lowest HbAc1 levels (7%), and the longest follow-up time (5.4 years) of all the trials analyzed (Table 3).

### FREEDOM-CVO

The FREEDOM-CVO safety trial was the fourth and final phase 3 clinical study of the FREEDOM program. It was a placebo-controlled cardiovascular outcomes study designed to meet the pre-approval safety assessment requirements set out in the U.S. Food and Drug Administration’s (FDA) Guidance for Industry to evaluate cardiovascular risk for new therapies to treat T2D. FREEDOM-CVO evaluated the safety of ITCA 650 at 60 µg per day vs. placebo in just over 4,000 patients on a variety of approved standard of care anti-diabetes therapies. ITCA 650 is a miniature osmotic pump system that is designed to deliver continuous subcutaneous release of exenatide. The duration of the study was dependent on event-based outcomes, and lasted just under 3 years, reaching the target number of cardiovascular events in the fourth quarter of 2015. The average treatment duration in FREEDOM-CVO was 1.2 years (ClinicalTrials.gov Identifier: NCT01455896). Age eligibility for the study was 40 years and older; other inclusion criteria stipulated that patients should have HbA1c > 6.5%, a history of coronary, cerebrovascular or peripheral artery disease, or multiple CV risk factors. There were a total of 160 strict MACE events observed in the FREEDOM-CVO trial. The overall safety and tolerability data for ITCA 650 was consistent with the three phase 3 trials that have already been presented and documented in the published literature for exenatide and other GLP-1 RA therapies [35–38]. ITCA submitted a new application to FDA for approval in October 2019. Non-inferiority was described but final results were not published so it in depth analysis will not be considered in this paper.

## Summary and discussion of the GLP-1 RA CVOTs results

All the GLP-1 RA CVOTs had an identical composite 3-point MACE primary outcome, including CV death, non-fatal MI or stroke (Table 1). The only exception was the ELIXA trial, which included an additional component of UA (4-point MACE) (Table 1). Nonetheless, there were different results obtained across the different trials, with some CVOTs showing superiority in the treatment arm, whilst others only demonstrated non-inferiority (Table 4). Even in trials which showed a positive effect, the significance of each component of the MACE outcome varied (see Table 4). This raises the question of whether or not we can consider the cardioprotective effects of GLP-1 RA as a class effect. It could in fact be the case that differences between drugs of this pharmacological class could explain the conflicting results, namely the long/short-action of the different GLP-1 RA and their molecule structure [39–41].

**Table 4:** Primary outcome results

	ELIXA [22]		LEADER [24]		SUSTAIN-6 [25]		EXSCEL [27, 28]		Harmony outcomes [30]		PIONEER 6 [32]		REWIND [34]	
	Lixisenatide	Placebo	Liraglutide	Placebo	Semaglutide	Placebo	Exenatide	Placebo	Albiglutide	Placebo	Semaglutide	Placebo	Dulaglutide	Placebo
N	3034	3034	4668	4672	1648	1649	7356	7396	4731	4732	1591	1592	4949	4952
MACE (per 100 patient-yr)	6.40	6.30	3.40^*^	3.90	3.24^*^	4.44	3.70	4.00	4.57^*^	5.87	2.90^*^	3.70	2.35^*^	2.66
CV death	2.30	2.40	1.20^*^	1.60	1.29	1.35	1.40	1.50	1.61	1.72	0.70	1.40	1.22	1.34
Non-fatal MI	4.20	4.10	1.60	1.80	1.40	1.92	(2.10) (fatal or nonfatal)	(2.10) (fatal or nonfatal)	(2.43) ^*^ (fatal or nonfatal)	(3.26) (fatal or nonfatal)	1.80	1.50	0.80	0.84
Non-fatal stroke	1.00	0.90	0.90	1.00	0.80^*^	1.31	(0.80) (fatal or nonfatal)	(0.90) (fatal or nonfatal)	(1.25) (fatal or nonfatal)	(1.45) (fatal or nonfatal)	0.60	0.80	0.52^*^	0.69
UA	0.20	0.10	(0.7)	(0.7)	(0.65)	(0.80)	N/A	N/A	N/A	N/A	(0.50)	(0.30)	(0.34)	(0.30)

*CV* cardiovascular, *MACE* major adverse cardiovascular outcome, *MI* myocardial infarction, *N/A* not applicable, *UA* unstable angina

^*^Statistically significant difference

Even though one cannot discard mere play of chance as the culprit, there are many other objective reasons for the differences observed. Besides drug tolerability and ease of administration, trial design and population can heavily influence the outcomes. As we describe, there are several important differences between the ways GLP-1 RA CVOTs were designed. For the REWIND [33] and EXSCEL [26] trials, the primary analysis aim was to show superiority, whereas for the others it was to show non-inferiority. Even for the non-inferiority trials, the upper limit of the 95% CI differed, with SUSTAIN-6 [25] and PIONEER 6 [31] using 1.8 rather than the more common 1.3 limit. Inclusion criteria also varied significantly, as we highlight above, as well as concomitant antidiabetic medication (Table 2).

The differences in trials design can be fully appreciated by analyzing the baseline population characteristics (Table 3). Trials using a higher upper limit for the 95% CI (SUSTAIN-6 [25] and PIONEER 6 [31]) had lower numbers of participants. The proportion of females was also different, being higher in the REWIND [33, 34] trial, that was specifically designed to include more women. Usually trials designed to evaluate CVO hat more male, because CV events are more frequent in males. There are also important differences in the mean baseline HbAc1 levels, as a consequence mainly of differences in inclusion/exclusion criteria. These trials were event-driven, and therefore their duration and mean follow-up times differ, varying from 1.3 (PIONEER 6 [32]) to 5.4 years (REWIND [34]), at least partially resulting from the different designs.

Perhaps most importantly, the trials differed substantially in the percentages of people with eCVD and/or CV risk factors. Most of them included a mixture of patients with eCVD and others with risk factors only, but the ELIXA [21, 22] and Harmony Outcomes [29, 30] trials had 100% of patients with eCVD, whereas the ESXCEL [26–28] trial included patients without CV risk factors.

An indirect way of assessing the risk of the population included in the different studies is to look at the event rate in the placebo group. In fact, in all the studies the event rate was higher than 2 per 100 patient-years (Table 4), which roughly corresponds to a 10-year event rate above 20%. We can conclude that we are always looking at very high-risk patients (formerly considered as coronary-risk equivalent [42]). Nonetheless, the event rate varied across the different trials, and seems to reflect differences in the population. For example, the lowest placebo event rate (2.7/100 patient-yr) was seen in the REWIND [34] trial, which is by far the one with the lowest percentage of patients with eCVD (31.5%), whereas the two highest placebo event rates were seen in the ELIXA [22] and Harmony Outcomes [30] trials (6.3 and 5.9/100 patient-yr, respectively), which were the only two with a population consisting exclusively of patients with eCVD.

A post-hoc analysis of the SUSTAIN-6 trial [43] showed that semaglutide reduces the risk of MACE in all the subgroups analyzed, which included grouping by age, gender, and CV risk. The duration of diabetes at baseline, however, seems to affect the outcomes, as demonstrated by a post-hoc analysis of the SUSTAIN-6 and LEADER trials [44]. A recent meta-analysis of all the discussed GLP-1 RA CVOTs found no significant heterogeneity across subgroups, which included primary versus secondary prevention, HbAc1 levels, length of follow-up, daily versus weekly administration, homology of the drug to human GLP-1, BMI, age, or baseline eGFR [45].

Nonetheless, the use of different definitions of eCVD in the different CVOTs that evaluated GLP-1 RA makes it difficult to compare results, even in a meta-analysis context; in fact, a patient-level meta-analysis would be needed, and that would involve data sharing between the research teams of all trials. This is of special importance due to the heterogeneity of the results regarding MACE and its different components, and raises questions about the generalizability of the results to the majority of the T2D population that does not have eCVD. Since reduction of cardiovascular disease occurred driven by reductions in either MI (HARMONY) [30], stroke (SUSTAIN-6 [25] and REWIND [34]) or CV death (LEADER) [24], it is of interest to understand if different drugs confer different benefits to patients. In this regard, PAD is relevant for several reasons: it frequently affects middle-aged and older patients with T2D [2] and the criteria for its presence were heterogeneous in the different studies. Furthermore, in the REWIND [34] trial PAD was only considered as present if patients had been submitted to revascularization. Bearing this in mind, it is conceivable that the same patient with PAD would have been included in the group without CVD in one study and in the one with CVD in the other. Considering that PAD may be the most frequent manifestation of CVD in patients with T2D [2], a careful interpretation of the available data should be sought, precluding a direct generalizability of the results found in trials with a high proportion of patients with eCVD.

Another approach to evaluate the generalizability of the results is to use the specific inclusion criteria of the different GLP-1RA CVOTs in the real-world setting, analyzing the proportion of patients that would fulfill inclusion criteria. This approach was used in different studies [46–48]. Overall, between 40 and 60% of the real-world diabetic population would meet the inclusion criteria. Of note, using the same real-world dataset, there was high heterogeneity between the proportion of patients that could have been included in different trials, emphasizing the problem of generalizability. However, a recent study evaluated the transposition of cardiovascular outcome trial effects to the real-world population using CVOT stratum-specific effects, showing that the cardiovascular benefits are transferable to a much different real-world population [49].

## Conclusions

We strongly believe that a fair and clinically useful comparison between the results of GLP-1 RA CVOTs discussed would involve post-hoc and meta-analyses in which the classification and grouping of patients would be done based on baseline clinical characteristics of each patient, which would then be grouped into different eCVD or CV risk factor classes designed according to the same definitions. Ideally, future CVOTs for antidiabetic drugs should be harmonized in such a way that consistent criteria are used across them, making comparisons between different drugs possible. This is particularly important in this new age of T2D management, in which personalized care based on patient’s characteristics should dictate the best drug therapy for each individual.

## Data Availability

Not applicable.
